# What can over 30 years of efforts to eradicate polio teach us about global health?

**DOI:** 10.1186/s12889-020-09198-z

**Published:** 2020-08-12

**Authors:** Olakunle Alonge

**Affiliations:** Department of International Health, Johns Hopkins Bloomberg School of Public Health, 615 N. Wolfe St, Baltimore, MD 21205 USA

The Global Polio Eradication Initiative (GPEI) – a global effort to eradicate polio (poliovirus and its debilitating diseases, e.g. severe forms of acute flaccid paralysis (AFP)) through vaccination and active surveillance – is the largest public health initiative in recent history. Since 1988, the program has delivered hundreds of billion doses of polio vaccines and vaccinated more than 2.5 billion children globally [1]. This effort has contributed to successfully wiping out cases of wild poliovirus (WPV) from almost every corner of the world, from 350,000 annual cases reported in 125 countries in 1988 to 143 cases in two countries in 2019 [2]. Endemic transmission of WPV now only occur in Afghanistan and Pakistan, and two of the three WPV strains (WPV2 and WPV3)^1^ have been declared eradicated as of 2019 [3, 4].

Human-to-human spread of poliovirus occurs at the individual level through the fecal-oral route, and as a result of interactions determined by human behaviors operating within specific social and physical environments. Given that human societies are complexly organized, global efforts to eliminate the spread of poliovirus are affected by a confluence of factors, e.g. politics, context-specific demand and supply-side factors, societal issues such as poverty and inequalities, and the social ills that results from these issues such as conflicts, insecurity and mistrust. Therefore, a successful polio eradication program requires collaboration among many stakeholders across multiple socioecological levels (individual, interpersonal, organizational, community and public policy), and governance and cooperation at global, regional, national and subnational levels. These challenges and criteria for success are not peculiar to the polio program but are the same for any efforts to confront a global disease (e.g. COVID-19, Ebola) or address health systems and other global health challenges for which all humans may be at risk. Hence, the polio program being the largest of its kind (in terms of scope, human and financial resources involved) provides an opportunity to learn important lessons about ‘how to’ and ‘how not to’ organize, strategize and implement activities to address global health challenges and achieve global health objectives.

To be clear, there is still a lot work to be done in order to stop the spread of poliovirus forever. The world is still very much at risk of an exponential rise in polio cases if ongoing efforts are not sustained until well after the eradication goal has been achieved. In 2014, polio was declared a public health emergency of international concern (PHEIC) by the World Health Organization (WHO) under the International Health Regulation (IHR) 2005 because there was evidence of exportation of poliovirus and cases internationally, within the WHO African and Eastern Mediterranean Regions, and from endemic countries to countries that have been polio-free for many years [5]. In January 2020, the status of polio as a PHEIC was preserved because of ongoing transmission of WPV1 within and between Pakistan and Afghanistan, increased incidence of new paralytic polio cases derived from the live attenuated (Sabin) viruses in the oral poliovirus vaccine (OPV), and evidence of widespread transmission of these vaccine-derived viruses (mainly type 2)^2^ among under-immunized populations in Africa and Asia [6, 7]. Between January 2018 and June 2019, outbreaks of circulating vaccine-derived polio viruses (cVDPV) (both type 1 and 2) were detected in more than 25 countries across 4 WHO regions (African, Eastern Mediterranean, South-East Asian and Western Pacific Regions [8]), and at least 350 cases of AFP due to cVDPV2 were reported in 13 African countries in 2019 alone [7]. Even after the spread of both wild and vaccine-derived polioviruses is stopped, ongoing containment efforts will be required to destroy stockpiles of vaccines containing live viruses and to ensure that secured polioviruses in laboratories all over the world are not re-introduced among the human population. The cost of failing to eradicate polio is astronomical, and best-case scenarios if only a global “control”^3^ is achieved (as opposed to an eradication^4^ goal) could result in as many as 200,000 new polio cases every year within 10 years [9–11]. Hence, discontinuing efforts towards achieving polio eradication is not option.

The ongoing spread of WPV1 in Afghanistan and Pakistan has been attributed to several factors, including: insecurity and ongoing conflicts which affect access to children for vaccination and surveillance, issues around refusals of individuals and communities to accept vaccination, and the politicization of the national polio program, especially in Pakistan [6, 12]. Similarly, multiple outbreaks of cVDPV have been attributed to ineffective coordination among actors, lack of timely vaccination campaigns in response to information from environmental surveillance data (which typically predates any outbreak of cVDPV cases) [12, 13], and weak routine immunization and health systems (which results in low vaccine coverage) [12, 14]. The GPEI leadership led by national governments with six core partners - the WHO, Rotary International, the US Centers for Disease Control and Prevention (CDC), the United Nations Children’s Fund (UNICEF), the Bill & Melinda Gates Foundation (BMGF) and, most recently, Gavi, the Vaccine Alliance – has outlined a systematic and coordinated plan to address these factors in the 2019–2023 GPEI Polio Endgame Strategy Plan [14]. Prior to the endgame strategy, the global community led by GPEI leadership had made considerable efforts, albeit less coordinated, to respond to some of these factors (and other issues with the polio program) as they arose. These efforts have yielded important benefits in many low and middle-income countries (LMICs), e.g. health workforce development, community engagement to build trust and social capital among hard-to-reach populations, integration of other vaccines and commodities to polio campaign and surveillance, strengthening of primary health care, provision of physical capital such as health facilities, laboratories, and vehicles for health services delivery, and establishment of mechanisms for coordination among diverse stakeholders in public health emergencies [15]. On the other hand, targeted efforts around the polio program, set against the backdrop of disenfranchisement and underdeveloped health and social services in certain communities, have raised suspicion among specific populations in LMICs which has contributed to refusals to accept polio vaccination among these communities [12].

The global community was aware of the risk of vaccine-associated paralytic polio (VAPP) from the use of the OPV as far back as the 1980s [16], prior to the launch of the GPEI in 1988. As of 2000, the Advisory Committee on Immunization Practices (ACIP) had recommended against the use of the OPV in the United States because it carried the risk of VAPP, but recommended the Inactivated Polio Vaccine (IPV) – with potency against WPV1, WPV2 and WPV3 and which does not contain any live viruses nor carry the risk of transmitting vaccine-derived polioviruses – be used instead [4]. Although IPV was discovered (in 1955) before OPV, the trivalent OPV became the vaccine of choice in many countries after it was introduced in 1963 because of the ease of oral administration, its ability to mimic natural poliovirus infection and induce immunity in the intestinal tracts (in addition to the bloodstream). Together, these attributes make OPV effective for rapidly controlling large polio epidemics through mass campaigns and by raising the herd immunity within large populations (i.e. limiting the number of susceptible individuals and the ability of the disease to spread  ) [4, 17]. The OPV may also confer passive immunity on non-vaccinated individuals via exposure to viruses being excreted by vaccinated individuals. However, given the known risk of VAPP and development of an enhanced-potency IPV in 1987 [4] with potential to confer superior herd immunity compared to OPV [17, 18], one can conclude from a purely scientific point of view that the ongoing problem with cVDPV could have been foreseen and adequate contingencies should have been made much earlier to rapidly expand the use of IPV globally. However, such decisions are not made easily. Aside from the argument about the limited production capacity of IPV to sustain a global program [17] – there are different incentive regimes faced by the multiple stakeholders directly involved in the GPEI that precludes such straightforward decision-making [19]. Unraveling these complex incentives in decision-making is key to advancing a global health agenda through alliances of multiple stakeholders. Although the GPEI leadership is ramping up efforts to roll out novel and stable strains of the OPV (which would be less likely to revert back to a virulent strain) to address the cVDPV outbreaks in different parts of the world, the large-scale effectiveness of these novel OPVs among human populations is yet to be seen [12, 20].

Given that the concerns about the polio program in places like Afghanistan and Pakistan are only being systematically addressed by the endgame strategy now, it can be concluded that perhaps the global community initially underestimated the significance of politics, societal norms, and context-specific demand and supply-side factors in shaping the success or failure of the polio eradication goal, while recognizing the importance of vaccine, the vaccine supply chain, campaign strategies to facilitate delivery of vaccines, and surveillance to track cases in real-time for dynamic decision-making. This may prove yet another important lesson for future global health programs and eradication activities, especially efforts to respond to global disease outbreaks and pandemics. In an interconnected world, we rise and fall together when faced with a disease outbreak – and the strength of any global emergency response system is as strong as its weakest link. Perhaps, coordinated efforts to understand and address these weak links should first be prioritized – and these efforts should include all relevant stakeholders as early as possible. Notably, the GPEI leadership only recently expanded to include Gavi, the Vaccine Alliance in 2019 [21] in recognition of the need to integrate efforts in polio with routine immunization in LMICs, and the 2019–2023 GPEI Polio Endgame Strategy Plan [14] is also the first time “integration” of polio with routine immunization and the broader health systems has become a main objective of the polio strategy with an associated action plan. In retrospect, the need to strengthen routine immunization to amplify and sustain the gains of polio program should have been foreseen and prioritized at the onset of the eradication efforts.

A major takeaway is that global health initiatives require a complex set of implementation activities – no matter how straightforward the health problem being addressed or evidence-based interventions that are specified for tackling the problem. However, there are key general factors that may determine the success or failure of such initiatives and these may play a significant role across diverse global health programs. A systematic study of the polio eradication program in different contexts provides the opportunity to identify this common set of general factors for advancing best practices in global health. The collection of articles under this journal supplement, titled *Lessons Learned from Global Polio Eradication*, describes lessons from the polio eradication experience to date based on data derived from a mixed method study conducted globally, and specifically in seven countries (Democratic Republic of Congo, Ethiopia, Nigeria, Afghanistan, Bangladesh, India, and Indonesia) representing WHO African, Eastern Mediterranean, and South-East Asian regions. The lessons are presented from an implementation science perspective [22, 23] and comprise of global and cross-country comparisons of implementation barriers and facilitators to polio eradication efforts to provide generalizable theories in global health for understanding implementation pathways, implementation strategies and their mechanisms across different contexts. These lessons and theories are relevant for addressing ongoing challenges with the GPEI (e.g. eliminating the risks of cVDPV) in specific contexts and transitioning resources of the GPEI to strengthen health systems in LMICs. Funders, policy-makers, and program managers may also apply these lessons and theories to understand critical bottlenecks and approaches for improving efficiency and effectiveness of other eradication and global control programs (e.g. measles, neglected tropical diseases, and malaria programs), and efforts to address global public health emergencies (e.g. outbreaks of Ebola Hemorrhagic Fever and 2019 coronavirus pandemic), especially in resource-limited settings where the polio eradication program continues to have a large-scale impact. The lessons could also be applied for teaching and research in core competencies of global health and implementation science by academics and researchers. Thus, over 30 years of efforts to eradicate polio can provide important roadmaps for improving health and achieving equity in health for all people worldwide.

## Data Availability

Not applicable.

## Abbreviations

ACIP: Advisory Committee on Immunization Practices
AFP: Acute Flaccid Paralysis
BMGF: Bill & Melinda Gates Foundation (BMGF)
CDC: U.S. Centers for Disease Control
cVDPV: Circulating Vaccine-Derived Poliovirus
GPEI: Global Polio Eradication Initiative
IHR: International Health Regulation
LMICs: Low and Middle-Income Countries
OPV: Oral Poliovirus Vaccine
PHEIC: Public Health Emergency of International Concern
UNICEF: United Nations Children’s Fund (UNICEF)
VAPP: Vaccine-Associated Paralytic Polio
WHO: World Health Organization
WPV: Wild Poliovirus

## Footnotes

1 *Wild type of poliovirus (WPV):* Wild type of poliovirus (WPV) is a single stranded RNA virus. It is a type of enterovirus from the family Picornavirdae. Humans are the only known reservoir for polio. There are 3 strains of wild type poliovirus (type 1, type 2, and type 3) – WPV1, WPV2, WPV3 (4).

2 *Emergence of cVDPV:* The trivalent oral polio vaccine (tOPV) consisted of live attenuated viruses (Sabin viruses – OPV1, OPV2, OPV3) that can elicit the production of protective antibodies against all 3 wild types. The OPV1, OPV2 and OPV3 could regain their virulence factor in rare cases to cause AFP and these virulent vaccine-derived viruses could be transmitted from person-to-person. The last case of WPV2 was detected in 1999, and OPV2 was dropped from the tOPV in 2016. However, monovalent OPV2 (mOPV2) continue to be used to respond to outbreaks of vaccine-derived poliovirus type 2 (VDPV2), and these vaccine-derived polioviruses may continue to circulate within populations with no or little polio immunization coverage.

3 *Disease control*: the reduction of disease incidence, prevalence, morbidity or mortality to a locally acceptable level as a result of deliberate efforts (i.e. continued intervention measures needed) (11).

4 *Disease eradication*: permanent reduction to zero of the worldwide incidence of infection caused by a specific agent as a result of deliberate efforts (continued intervention measures are no longer needed) (11).
